# Post-Endoscopic Retrograde Cholangiopancreatography Pancreatitis: What We Already Know

**DOI:** 10.7759/cureus.21773

**Published:** 2022-01-31

**Authors:** Adham E Obeidat, Ratib Mahfouz, Gabriel Monti, Landon Kozai, Mohammad Darweesh, Mahmoud M Mansour, Ahmad Alqam, David Hernandez

**Affiliations:** 1 Internal Medicine, University of Hawaii, Honolulu, USA; 2 Internal Medicine, Kent Hospital, Warwick, USA; 3 Internal Medicine, Oregon Health & Science University, Portland, USA; 4 Internal Medicine, East Tennessee State University, Johnson City, USA; 5 Internal Medicine, University of Missouri School of Medicine, Columbia, USA; 6 Internal Medicine, Englewood hospital, Englewood, USA; 7 Internal Medicine, Brown University, Providence, USA

**Keywords:** lipase, ercp, amylase, acute pancreatitis, abdominal pain

## Abstract

Acute pancreatitis is the most common serious complication of endoscopic retrograde cholangiopancreatography (ERCP) resulting in significant morbidity and occasional mortality. Post-ERCP pancreatitis (PEP) has been recognized since ERCP was first performed, and many studies have shown a consistent risk that must be balanced against the many benefits of this procedure. This review will discuss the pathogenesis, epidemiology, potential risk factors, and clinical presentation of PEP. Moreover, it will discuss in detail the most recent updates of PEP prevention and management.

## Introduction and background

Since the introduction of endoscopic retrograde cholangiopancreatography (ERCP) in 1968, it has been used as a diagnostic and therapeutic procedure for multiple biliary and pancreatic diseases. Acute pancreatitis is the most common serious complication of ERCP resulting in significant morbidity and occasional mortality and accounting for more than $200 million of health expenditure annually [1]. Post-ERCP pancreatitis (PEP) has been recognized since ERCP was first performed, and numerous studies over the ensuing decades have shown a consistent risk that must be balanced against the many benefits of the procedure. This review will describe the pathogenesis, epidemiology, risk factors, clinical presentation, diagnosis, treatment, and prevention of PEP as it is known today.

## Review

Definition and pathogenesis

PEP was originally defined as a clinical syndrome of abdominal pain and elevated serum amylase, at least three times the upper level of normal, which occurs more than one week after ERCP for any reason [1-3]. Freeman et al. proposed using lipase as a possible alternative to amylase and defining clinical pancreatitis as a new or worsened abdominal pain [4]. According to the Atlanta classification of acute pancreatitis, which was updated in 2012, the diagnosis of PEP requires two of the following criteria: abdominal pain, serum lipase or amylase at least three times greater than the upper level of normal, and characteristic findings of acute pancreatitis on computed tomography (CT), magnetic resonance imaging (MRI), or transabdominal ultrasonography [3,5].

There are two major mechanisms by which ERCP can cause injury and subsequent pancreatic inflammation: mechanical damage to the pancreatic duct or hydrostatic insult from contrast [6-9]. Many ERCP procedures require prolonged or repeated instrumentation of the pancreatic duct, which can result in direct trauma to the duct or ampulla. Similarly, the use of electrocautery can result in thermo-injury to these same structures. This can result in reactive edema following ERCP, which can lead to pancreatic duct obstruction and impaired emptying of enzymatically active pancreatic secretions [6,9].

The role of contrast agents and hydrostatic injury to the pancreatic duct is controversial. Overuse of contrast during ERCP can occur, and the mechanism by which contrast induces damage to the duct could be either chemical or allergic, though the rate of PEP does not seem to differ depending on the type of contrast used, even when comparing high and iso-osmolar agents [6-9].

Other potential mechanisms such as direct enzymatic activation; genetic abnormalities, in particular, homozygous alpha-1-anti trypsin deficiency; and bacterial seeding have also been suggested. However, these appear to contribute less than either mechanical or contrast-induced injury [6,9,10].

Epidemiology and risk factors

PEP is the most common complication of ERCP, with an incidence rate that ranges between 2% and 5% [11-20]. Rates of severe pancreatitis are notably less, typically less than 0.5%. A systematic review by Kochar et al. that examined more than 13,296 patients who underwent ERCP with no prophylactic drugs or stent placement showed an overall incidence of 9.7% and a mortality rate of 0.7%. It also showed a minor difference between incidence in North America (13%) as compared to Europe (8.4%) and Asia (9.9%) [16]. Moreover, another systematic review by Andriulli et al., which included 21 studies involving 16,855 patients, found a 3.5% incidence of PEP, of which 0.4% of patients had severe pancreatitis, and a mortality rate of 0.11% [11].

Numerous modifiable and non-modifiable risk factors have been implicated in the development of PEP (Table 1). These risk factors can be patient-related, endoscopist-related, or procedure-related [9,13,21,22]. It has been suggested that these risk factors are additive, meaning that patients with numerous risk factors should be evaluated with caution as they can be at an increased risk of PEP. It has been even suggested that the presence of one or more independent risk factors is reason enough to justify an overnight stay for post-ERCP patients [9,10].

**Table 1:** Risk factors for post-ERCP pancreatitis ARB, angiotensin receptor blocker; ERCP, endoscopic retrograde cholangiopancreatography; IPMN, intraductal papillary mucinous neoplasm; PEP, post-ERCP pancreatitis

Stronger Evidence	Weaker or Conflicting Evidence
Female gender	Younger age
Prior history of PEP	Operator inexperience
Prior history of pancreatitis	Normal serum bilirubin
Endoscopic sphincterotomy	Therapeutic ERCP (as compared to diagnostic ERCP)
Sphincter of Oddi dysfunction	ARB use
Placement of non-prophylactic pancreatic duct stent	Smoking
IPMN	Low medical center case volume
Main pancreatic duct contrast injection	Metallic stent (as compared to plastic)
Difficult cannulation	Papillary balloon dilation
	Retained biliary duct stones

Chen et al. have reported in a meta-analysis of 13 studies involving 32,381 post-ERCP patients that female gender, previous PEP, previous pancreatitis, endoscopic sphincterotomy, precut sphincterotomy, sphincter of Oddi dysfunction (SOD), and non-prophylactic pancreatic duct stent are significantly associated with an increased risk of PEP [12]. Furthermore, Ding et al. in a meta-analysis of 28 studies involving 54,889 patients concluded that in addition to the previously mentioned factors, intraductal papillary mucinous neoplasm, difficult cannulation, and main pancreatic duct injection were also associated with an increased risk of PEP [14]. In fact, there is some controversy about whether precut sphincterotomy is an independent risk factor for PEP or whether multiple cannulation attempts with subsequent papillary trauma is actually to blame. Other risk factors that have been also reported include younger age, operator lack of experience, normal serum bilirubin, and the use of therapeutic ERCP [11,12,15,17,18].

There are numerous other risk factors that have been described but not proven to contribute to the development of PEP. Li et al. in a retrospective analysis of 506 patients who underwent ERCP found that the use of angiotensin receptor blockers, smoking, and younger age were independent risk factors for PEP, while propofol was a safe sedative agent for ERCP and associated with no increased risk of PEP [23]. Testoni et al. showed in a prospective study of 3,635 patients who underwent ERCP that the risk of PEP did not differ between high and low-volume centers (3.9% vs. 3.1%) or between expert and non-expert operators (3.8% vs. 5.5%) [22]. On the other hand, another prospective study of ERCP procedures performed in 28 centers in France showed that the success of ERCP and post-ERCP morbidity is related to center activity level and operator experience [24].

Moreover, Wilcox et al. described that bile duct stent placement was an independent risk factor for PEP while performing sphincterotomy before stenting, and stent characteristics had no association [25]. However, another retrospective analysis of 544 patients who underwent ERCP for malignant biliary obstruction found that the frequency of PEP was significantly higher with the placement of a metallic stent compared to a plastic stent. In this study, rates of PEP were comparable with the use of covered and uncovered metallic stents [26].

The Japanese Society of Gastroenterology considers the following as risk factors for PEP: SOD, female sex, history of pancreatitis, precutting procedures, and contrast injection of the pancreatic duct. On the other hand, young age, absence of extrahepatic bile duct dilation, normal serum bilirubin, more than five cannulation attempts, pancreatic sphincterotomy, papillary balloon dilation, and residual bile duct stones are considered factors that physicians should pay more attention to for the possibility of PEP [27].

Clinical presentation and diagnosis

The presentation of PEP is identical to acute pancreatitis of other etiologies. The cardinal symptoms and signs include epigastric pain, abdominal tenderness, and elevated serum amylase and/or lipase. The European Society of Gastrointestinal Endoscopy (ESGE) 2014 guidelines recommends checking serum amylase or lipase two to six hours after the procedure in patients who have symptoms. Patients with amylase or lipase value less than 1.5 and four times the upper normal limit, respectively, can be discharged without concern about the risk of PEP. However, it can be challenging to differentiate PEP from transient abdominal pain and elevated serum amylase from intestinal distention within 24 hours of ERCP [3,10].

Various diagnostic criteria for PEP have been suggested. Testoni et al. concluded that serum amylase level measured four hours after endoscopic sphincterotomy was a reliable indicator of PEP. They found that more than two-thirds of PEP cases occurred among the patients whose four-hour amylase level was higher than five times the normal upper limit [10,28]. Ito et al. suggested that a dynamic rise of serum amylase between three to six hours post-procedure can be diagnostic of PEP. They suggested that when serum amylase level higher than two times the normal upper limit is observed at three hours after ERCP, serum amylase level should be repeated six hours after the procedure. A drop in serum amylase level at six hours post-ERCP indicates an absence of PEP [29]. One single-center observational study by Amornyotin et al. has proposed to use the intensity of patients’ pain in the first six hours after ERCP by using visual analog scales as a diagnostic tool for PEP [30].

Other alternative biochemical markers for PEP diagnosis have been suggested by small observational series. Among the markers that were proven to be associated with PEP were trypsinogen, trypsinogen activation peptide, C-reactive protein, serum elastase-1, erythrocyte sedimentation rate, chemerin, and various interleukins (ILs) such as IL-6 and IL-10 [31-36].

The severity of PEP can be classified based on the length of hospital admission and the need for intervention. It can be divided into mild, moderate, and severe (Table 2) [2]. Another classification, the Atlanta classification, is based on complications, in particular organ failure, though it is used to describe all acute pancreatitis cases, not just PEP [5]. The Atlanta classification, which was revised in 2012, defines mild acute pancreatitis as involving no organ failure, local complications, or systemic complications. On the other hand, moderate acute pancreatitis is defined as having organ failure that resolves within 48 hours and local or systemic complications that do not cause persistent organ failure, while severe acute pancreatitis is defined as persistent single or multiple organ failures >48 hours (Table 2).

**Table 2:** Severity classification for acute pancreatitis ERCP, endoscopic retrograde cholangiopancreatography; PEP, post-ERCP pancreatitis

Severity	Mild PEP	Moderate PEP	Severe PEP
Serum amylase Level (>24 hours post-ERCP)	>3x upper limit of normal	>3x upper limit of normal	>3x upper limit of normal
Separate admission or extension of hospitalization	2-3 Days	4-10 days	>10 days
Complications			Hemorrhagic pancreatitis, phlegmon, or pseudocyst
Need for intervention			Requires drainage or surgical intervention

Treatment

As not all patients with abdominal pain and elevated amylase or lipase levels post-ERCP have PEP, this makes it harder for clinicians to identify patients with PEP, and thus it makes it harder to initiate treatment at the early stages of the disease. Management of PEP is similar to acute pancreatitis from other etiologies, which is mainly supportive with aggressive IV fluid hydration and pain control [37,38]. Early and aggressive fluid resuscitation appears to decrease the risk of systemic inflammatory response syndrome and organ failure [37,39]. Wu et al. proposed that lactated Ringer, which is a more pH-balanced fluid, may be less likely to stimulate inflammatory mediators that function better in an acidic environment [40].

Close monitoring for signs and symptoms of organ dysfunction is critical, as a mild PEP can sometimes progress into a life-threatening necrotizing disease. Moreover, early identification of patients with severe PEP is crucial, as such patients may require management in the intensive care unit. Nutrition and multidisciplinary management of complications such as necrosis and abscesses are critical [37].

Prevention

As pancreatitis is still considered to be the most common serious side effect of ERCP resulting in significant morbidity and mortality, preventive and prophylactic measures were studied extensively. Here we summarize the most recent data on how to prevent PEP.

Patient Selection

Careful patients’ selection whether to undergo ERCP or not remains the most important prevention strategy. Magnetic resonance cholangiopancreatography (MRCP) and endoscopic ultrasound (EUS) provide highly accurate imaging of the pancreaticobiliary system that can eliminate the need for ERCP [9]. EUS has a sensitivity of 89.5%, specificity of 96.5%, and a positive predictive value of 91.9% in diagnosing biliary obstruction compared to ERCP as a gold standard. It is generally recommended to proceed with ERCP only after negative EUS and persistent suspicion of obstructed biliary duct to minimize such invasive procedure [40]. In the EPISOD trial, ERCP with manometry and sphincterotomy has not been found to reduce disability due to abdominal pain after cholecystectomy, most likely due to SOD, which strongly affected the indication for ERCP in such patients [41]. Therefore, the use of ERCP is moving toward becoming an exclusively therapeutic procedure, as there are now less invasive but nearly accurate diagnostic tests.

Risk Stratification

Patient-related and procedure-related risk factors are described in Table 1. These risk factors were found to be independent based on multivariate analysis and can increase PEP’s rate synergistically with a cumulative effect [9]. It is thought that the highest risk of PEP (>40%) was found in females with a SOD, normal serum bilirubin level, and difficult biliary cannulation [42]. On the other hand, some clinical characteristics are thought to reduce the risk of PEP. Chronic pancreatitis may decrease the risk of developing PEP because of gland fibrosis, atrophy, and reduced enzyme activity. Moreover, age would also affect pancreatic function and decrease the risk of PEP. Furthermore, biliary interventions with previous biliary sphincterotomy would decrease the risk as it will separate the biliary and pancreatic orifices with less risk of trauma to the pancreatic duct [42]. Finally, the presence of malignant biliary obstruction caused by pancreatic cancer was not associated with an PEP. This is because the pancreatic duct is already blocked and there is already significant ductal and parenchymal damage [43].

A bedside scoring system was suggested to estimate the risk of developing PEP, which includes pain during the procedure (4 points), cannulation of the pancreatic duct (3 points), previous PEP (2 points), and the number of cannulation attempts (1-4 points depending on the number of attempts). A total score of 1-4 points is associated with a low risk of pancreatitis (<2%), a score of 5-8 points has an intermediate risk (7%), and a score of 9 or above is associated with high risk (28%) [44].

Endoscopic Techniques

A number of techniques and approaches can be used to decrease the probability of developing PEP, including careful use of electrocautery current during sphincterotomy, prophylactic pancreatic stent (PPS) placement in patients who are at a high risk of developing PEP, and wire-guided techniques for deep biliary cannulation.

Electrocautery: The application of electrosurgical current during biliary or pancreatic sphincterotomy can contribute to causing PEP through thermal injury [9]. The risk of PEP can be influenced by the type of electrocautery used for sphincterotomy. However, studies have reached variable conclusions. In certain studies, the pure cutting current has been shown to reduce the incidence of PEP compared to blended cutting currents (3.5% vs 12%) [45]. Other studies suggested less risk with bipolar compared with the standard monopolar electrocautery (0% vs 12%) [46]. However, four trials were included in a meta-analysis with a total of 804 patients to compare pure cutting current with blended current as a technique for sphincterotomy, with the conclusion of no significant difference in the incidence of PEP [47]. Some endoscopists prefer to begin the sphincterotomy with a pure cutting current and finish it with a blended current. However, studies could not prove its effectiveness in decreasing the PEP incidence [47,48]. In addition, the automatic variable intensity of current and current blend according to tissue resistance was not found to decrease the risk of PEP [49].

Pancreatic stenting: Patients who are at a high risk of PEP may benefit from PPS. It is hypothesized to reduce the intraductal pancreatic pressure from papillary edema and stenosis [7]. In a meta-analysis of 14 studies, PPS was associated with a statistically significant reduction of PEP (RR = 0.39) [50]. Another meta-analysis has reported a decrease in odds of PEP with PPS (OR = 0.22) [51]. PPS was also shown to decrease the incidence and likelihood of severe and necrotizing pancreatitis [50,51].

Despite its benefits, PPS is not risk-free and has its own potential complications such as stent migration, cholangitis or cholecystitis, bleeding, infection, occlusion, and perforation, which can occur in up to 4.4% of cases [52]. A recent meta-analysis included six randomized controlled trials (RCTs) and concluded that 5-Fr pancreatic stent is superior to the 3-Fr pancreatic stent in preventing PEP. The stent diameter was far more important in prevention than the type of stent or the presence of flanges [53]. Another study revealed a superiority of 3-cm stents compared with 5-cm stents [54]. In conclusion, 5-Fr, 3-cm stents are associated with the least risk of PEP compared to other lengths and diameters.

Cannulation techniques: Interventions that can improve cannulation efficiency and limit contrast injection into the pancreas are thought to reduce the risk of PEP. A Cochrane meta-analysis of 12 RCTs involving 3,450 patients found that PEP incidence was lower in the wire-guided cannulation group (3.5%) compared to contrast-assisted cannulation technique (6.7%) and that primary cannulation rates were higher as well (84% vs 77%, RR = 1.07). However, wire-guided cannulation may not prevent PEP in patients with suspected SOD dysfunction and unintentional pancreatic duct guidewire cannulation [55]. Another meta-analysis showed that guidewire cannulation was associated with lower PEP rates (0%-3%) compared to the standard contrast-injection method (4%-12%) and increased primary cannulation rates compared to the standard method (OR = 2.05) [56].

However, other studies have failed to confirm the protective benefit of wire-guided cannulation [57,58]. Moreover, one study revealed that unintentional guidewire insertion into the pancreatic duct or a small common bile duct (<9 mm in diameter) can be a risk factor for PEP [59]. Occasionally, the double wire approach is used when initial cannulation attempts result in the wire being accidentally passed into the pancreas. The wire is left in the pancreatic duct so that it straightens the common channel, partially obstructs the pancreatic orifice, and provides a fluoroscopic reference for subsequent biliary access. Unfortunately, the use of the double-guidewire technique did not lead to a statistically significant decrease in PEP incidence. Those with malignant biliary stricture and other anatomic abnormalities were more likely to benefit from double-guidewire cannulation [60]. Pancreatic duct perforation is by far the most significant risk when wire-guided cannulations are used.

Intravenous Hydration With Lactated Ringer

The American Society for Gastrointestinal Endoscopy recommends the use of periprocedural intravenous hydration with lactated Ringer to reduce the risk of PEP [61]. A meta-analysis of nine RCTs that included 2,094 patients concluded that aggressive hydration with lactated Ringer decreases the incidence of PEP by 56% compared to standard hydration. In addition, it decreases the length of stay by one day, with no significant difference in fluid overload complications [62]. Aggressive hydration strategy with an initial fluid rate of 3 cc/kg/hour during the procedure and 20 cc/kg bolus immediately after the procedure, in addition to 3 cc/kg/hour for eight hours following the procedure, seems a good fluid rate as evident among studies in which aggressive hydration is preferred [39,62].

Chemoprevention

Several classes of drugs have been studied to prevent PEP, including anti-inflammatory drugs, protease inhibitors, pancreatic enzymes, drugs that decrease sphincter of Oddi pressure, antibiotics, antioxidants, and anti-metabolites. Only rectally administered non-steroidal anti-inflammatory drugs (NSAIDs) have been proven to decrease the incidence.

Anti-inflammatory drugs: The mechanism of NSAIDs is thought to be related to their potency in inhibiting phospholipase A2, which appears to play a significant role in initiating the inflammatory cascade that leads to pancreatitis. It was found that indomethacin followed by diclofenac were the most potent inhibitors of phospholipase A2 [63]. Based on a meta-analysis of 17 RCTs, PEP incidence fell significantly with the use of diclofenac or indomethacin, and they were even superior to pancreatic duct stents in preventing PEP. However, indomethacin or diclofenac administered by other means were not as effective as the rectal route, as it has a higher bioavailability compared to other routes, with significant first-pass metabolism and higher peak plasma levels. The efficacy of indomethacin and diclofenac were similar in general whether given pre- or post-procedure or whether in average or high-risk patients. Nonetheless, in only high-risk patients, indomethacin pre-procedure administration was more effective in reducing the risk of PEP when compared with post-procedure administration [64,65].

Glucocorticoids have been studied due to their potent anti-inflammatory effect. The results of a meta-analysis of six RCTs investigating the use of intravenous or oral corticosteroids showed that prophylactic corticosteroids had no benefit in reducing PEP [66]. Moreover, IL-10 has been found to reduce the severity of acute pancreatitis in animal models due to its anti-inflammatory effects. A study that included 144 patients found that a single intravenous dose given 30 minutes before ERCP reduced the risk of pancreatitis [67]. However, two other controlled trials involving a total of 505 patients failed to show any associated benefit [68,69]. Allopurinol was studied as well and was found to inhibit the production of oxygen-derived radicals. According to two meta-analyses of 10 RCTs, allopurinol was not effective in reducing PEP [70,71].

Protease inhibitors: Protease inhibitors such as nafamostat, gabexate, and ulinastatin have been investigated as well, as protease is thought to be contributing to the pathogenesis of PEP. Studies have shown mixed results, as some showed a reduction of PEP incidence, while others did not show any benefit, especially in patients with high risk. A meta-analysis of 18 studies did not find any evidence to support using protease inhibitors to decrease PEP incidence [71]. However, a more recent study concluded that unlike gabexate and ulinastatin, a combination of NSAIDs and nafamostat can decrease the incidence of PEP [72].

There has been a controversy over the use of prophylactic heparin in the prevention of PEP; however, a meta-analysis of four studies demonstrated no associated benefit [73]. Increased calcium concentration in the pancreatic endocrine system plays a central role in initiating intracellular protease activation, a critical step in acute pancreatitis. And as magnesium can act as a calcium antagonist that inhibits calcium signaling, researchers are investigating whether intravenous magnesium sulfate can be used to prevent PEP [74].

Other pancreatic enzymes inhibitors: Somatostatin efficacy has been studied in multiple trials with various conclusions. A meta-analysis of nine studies concluded that short-term (6 hours) and long-term (12 hours) administration of somatostatin was ineffective in preventing PEP [65]. Another meta-analysis showed no benefit of somatostatin when given as a short-term infusion, but it was effective when given as a single bolus or as a longer-term infusion [75]. Another recent meta-analysis confirmed the efficacy of somatostatin in PEP prophylaxis as a long-term infusion [66]. Compared with somatostatin alone, a combination of diclofenac and somatostatin was more effective in preventing the development of PEP [67]. However, as of now, somatostatin and octreotide are not officially recommended yet to be used in PEP prevention. On the other hand, calcitonin was studied and was not found to affect PEP incidence [76].

Reduction in the sphincter of Oddi pressure: Nitroglycerine was found to reduce sphincter of Oddi pressure, as it inhibits the contraction of smooth muscles, relieving the sphincter, and also enhances blood flow to the pancreas [77]. Initial studies found that nitroglycerine can decrease the incidence of PEP compared to placebo [78,79]. On the other hand, subsequent studies failed to show any associated benefit [80,81]. However, a meta-analysis conducted in 2010 showed that nitroglycerine can decrease the incidence of PEP [68]. The combination of nitroglycerin with NSAIDs provided more benefits than either of them alone [71]. Although there is little evidence to support nitrate efficacy, it can still be a potential prophylactic option in those who are contraindicated to NSAIDs.

Secretin increases pancreatic secretion and relaxes the sphincter of Oddi, and it has been found to lower the incidence of pancreatitis (8.7% vs 15.1%) in patients who had undergone a biliary sphincterotomy [82]. On the other hand, botulinum toxin, lidocaine, phosphodiesterase inhibitor type 5, and nifedipine were not proven to be useful [83-88].

Antibiotics: A prospective study of 321 patients found that 2 g of ceftazidime 30 minutes before ERCP can significantly reduce the incidence of PEP. These results suggest that bacteria could contribute to the pathogenesis of PEP. Therefore, antibiotic prophylaxis can be routinely recommended prior to ERCP, but it still needs further investigations [89].

Antioxidants and antimetabolites: Beta-carotene has been studied in a double-blinded trial, which showed no difference in the incidence of pancreatitis between the treatment group and the placebo group. However, the study suggested a possible protective effect regarding the severity of the disease, as no patients in the beta-carotene group had severe pancreatitis [90]. A recent meta-analysis including 11 RCTs studied the benefits of N-acetylcysteine, selenite, beta-carotene, allopurinol, and pentoxifylline in preventing PEP and concluded that antioxidative supplements do not have a beneficial effect in reducing the incidence of PEP [91]. On the other hand, a prospective case-control study of 160 patients concluded that 5-FU added in meglumine diatrizoate can decrease the incidence of PEP and hyperamylasemia [92].

## Conclusions

Acute pancreatitis is a common complication of ERCP and results in significant morbidity and occasional mortality. The risk of this complication must be weighed against the benefit of performing this procedure. Numerous risk factors for this complication have been identified and should be considered prior to performing this procedure. The diagnosis of PEP may be established using clinical, laboratory, and radiographic data, although several diagnostic criteria have been proposed. However, this complication may be difficult to detect due to the variability of its presentation. When suspected, treatment should begin promptly with close monitoring for deterioration. PEP may be prevented with careful patient selection, risk stratification, endoscopy techniques, and medications.

## References

[1] Tenner S, Baillie J, DeWitt J, Vege SS (2013). American College of Gastroenterology guideline: management of acute pancreatitis. Am J Gastroenterol.

[2] Cotton PB, Lehman G, Vennes J (1991). Endoscopic sphincterotomy complications and their management: an attempt at consensus. Gastrointest Endosc.

[3] Dumonceau JM, Andriulli A, Elmunzer BJ (2014). Prophylaxis of post-ERCP pancreatitis: European Society of Gastrointestinal Endoscopy (ESGE) Guideline - updated June 2014. Endoscopy.

[4] Freeman ML, Nelson DB, Sherman S (1996). Complications of endoscopic biliary sphincterotomy. N Engl J Med.

[5] Banks PA, Bollen TL, Dervenis C (2013). Classification of acute pancreatitis--2012: revision of the Atlanta classification and definitions by international consensus. Gut.

[6] Sherman S, Lehman GA (1991). ERCP- and endoscopic sphincterotomy-induced pancreatitis. Pancreas.

[7] Donnellan F, Byrne MF (2012). Prevention of post-ERCP pancreatitis. Gastroenterol Res Pract.

[8] Freeman ML, Guda NM (2004). Prevention of post-ERCP pancreatitis: a comprehensive review. Gastrointest Endosc.

[9] Wong LL, Tsai HH (2014). Prevention of post-ERCP pancreatitis. World J Gastrointest Pathophysiol.

[10] Tryliskyy Y, Bryce GJ (2018). Post-ERCP pancreatitis: pathophysiology, early identification and risk stratification. Adv Clin Exp Med.

[11] Andriulli A, Loperfido S, Napolitano G (2007). Incidence rates of post-ERCP complications: a systematic survey of prospective studies. Am J Gastroenterol.

[12] Chen JJ, Wang XM, Liu XQ (2014). Risk factors for post-ERCP pancreatitis: a systematic review of clinical trials with a large sample size in the past 10 years. Eur J Med Res.

[13] Cheng CL, Sherman S, Watkins JL (2006). Risk factors for post-ERCP pancreatitis: a prospective multicenter study. Am J Gastroenterol.

[14] Ding X, Zhang F, Wang Y (2015). Risk factors for post-ERCP pancreatitis: a systematic review and meta-analysis. Surgeon.

[15] Finkelmeier F, Tal A, Ajouaou M, Filmann N, Zeuzem S, Waidmann O, Albert J (2015). ERCP in elderly patients: increased risk of sedation adverse events but low frequency of post-ERCP pancreatitis. Gastrointest Endosc.

[16] Kochar B, Akshintala VS, Afghani E (2015). Incidence, severity, and mortality of post-ERCP pancreatitis: a systematic review by using randomized, controlled trials. Gastrointest Endosc.

[17] Maitin-Casalis N, Neeman T, Thomson A (2015). Protective effect of advanced age on post-ERCP pancreatitis and unplanned hospitalisation. Intern Med J.

[18] Yaghoobi M, Pauls Q, Durkalski V (2015). Incidence and predictors of post-ERCP pancreatitis in patients with suspected sphincter of Oddi dysfunction undergoing biliary or dual sphincterotomy: results from the EPISOD prospective multicenter randomized sham-controlled study. Endoscopy.

[19] Cheon YK, Cho KB, Watkins JL, McHenry L, Fogel EL, Sherman S, Lehman GA (2007). Frequency and severity of post-ERCP pancreatitis correlated with extent of pancreatic ductal opacification. Gastrointest Endosc.

[20] Glomsaker T, Hoff G, Kvaløy JT, Søreide K, Aabakken L, Søreide JA (2013). Patterns and predictive factors of complications after endoscopic retrograde cholangiopancreatography. Br J Surg.

[21] Cotton PB, Garrow DA, Gallagher J, Romagnuolo J (2009). Risk factors for complications after ERCP: a multivariate analysis of 11,497 procedures over 12 years. Gastrointest Endosc.

[22] Testoni PA, Mariani A, Giussani A (2010). Risk factors for post-ERCP pancreatitis in high- and low-volume centers and among expert and non-expert operators: a prospective multicenter study. Am J Gastroenterol.

[23] Li N, Tieng A, Novak S (2010). Effects of medications on post-endoscopic retrograde cholangiopancreatography pancreatitis. Pancreatology.

[24] Vitte RL, Morfoisse JJ (2007). Evaluation of endoscopic retrograde cholangiopancreatography procedures performed in general hospitals in France. Gastroenterol Clin Biol.

[25] Wilcox CM, Phadnis M, Varadarajulu S (2010). Biliary stent placement is associated with post-ERCP pancreatitis. Gastrointest Endosc.

[26] Coté GA, Kumar N, Ansstas M, Edmundowicz SA, Jonnalagadda S, Mullady DK, Azar RR (2010). Risk of post-ERCP pancreatitis with placement of self-expandable metallic stents. Gastrointest Endosc.

[27] Mine T, Morizane T, Kawaguchi Y (2017). Clinical practice guideline for post-ERCP pancreatitis. J Gastroenterol.

[28] Testoni PA, Bagnolo F, Caporuscio S, Lella F (1999). Serum amylase measured four hours after endoscopic sphincterotomy is a reliable predictor of postprocedure pancreatitis. Am J Gastroenterol.

[29] Ito K, Fujita N, Noda Y, Kobayashi G, Horaguchi J, Takasawa O, Obana T (2007). Relationship between post-ERCP pancreatitis and the change of serum amylase level after the procedure. World J Gastroenterol.

[30] Amornyotin S, Phasurin T, Wongnuch P (2009). Pain score within twenty-four hours post-endoscopic retrograde cholangiopancreatography: a comparison between diagnostic and therapeutic procedures. Gastroenterol Insights.

[31] Devière J, Le Moine O, Van Laethem JL, Eisendrath P, Ghilain A, Severs N, Cohard M (2001). Interleukin 10 reduces the incidence of pancreatitis after therapeutic endoscopic retrograde cholangiopancreatography. Gastroenterology.

[32] Katsanos KH, Tzambouras N, Baltayiannis G (2002). The true value of serum elastase-1 in endoscopic retrograde cholangiopancreatography (ERCP). Eur J Intern Med.

[33] Jin T, Huang W, Jiang K (2013). Urinary trypsinogen-2 for diagnosing acute pancreatitis: a meta-analysis. Hepatobiliary Pancreat Dis Int.

[34] Sultan S, Baillie J (2002). What are the predictors of post-ERCP pancreatitis, and how useful are they?. JOP.

[35] Mohammad Alizadeh AH, Afzali ES, Behzad C, Mousavi M, Mirsattari D, Doagoo SZ, Zali MR (2015). Is ESR Important for predicting post-ERCP pancreatitis?. Clin Med Insights Gastroenterol.

[36] Koksal AR, Boga S, Alkim H, Sen I, Neijmann ST, Alkim C (2016). Chemerin: a new biomarker to predict postendoscopic retrograde cholangiopancreatography pancreatitis. Eur J Gastroenterol Hepatol.

[37] Sahakian AB, Buxbaum JL, Van Dam J (2021). Prevention and management of post-ERCP pancreatitis. JOP.

[38] Gardner TB, Vege SS, Chari ST (2009). Faster rate of initial fluid resuscitation in severe acute pancreatitis diminishes in-hospital mortality. Pancreatology.

[39] Wu BU, Hwang JQ, Gardner TH (2011). Lactated Ringer's solution reduces systemic inflammation compared with saline in patients with acute pancreatitis. Clin Gastroenterol Hepatol.

[40] Anwer M, Asghar MS, Rahman S (2020). Diagnostic accuracy of endoscopic ultrasonography versus the gold standard endoscopic retrograde cholangiopancreatography in detecting common bile duct stones. Cureus.

[41] Cotton PB, Durkalski V, Romagnuolo J (2014). Effect of endoscopic sphincterotomy for suspected sphincter of Oddi dysfunction on pain-related disability following cholecystectomy: the EPISOD randomized clinical trial. JAMA.

[42] Freeman ML, DiSario JA, Nelson DB (2001). Risk factors for post-ERCP pancreatitis: a prospective, multicenter study. Gastrointest Endosc.

[43] Banerjee N, Hilden K, Baron TH, Adler DG (2011). Endoscopic biliary sphincterotomy is not required for transpapillary SEMS placement for biliary obstruction. Dig Dis Sci.

[44] Friedland S, Soetikno RM, Vandervoort J, Montes H, Tham T, Carr-Locke DL (2002). Bedside scoring system to predict the risk of developing pancreatitis following ERCP. Endoscopy.

[45] Elta GH, Barnett JL, Wille RT, Brown KA, Chey WD, Scheiman JM (1998). Pure cut electrocautery current for sphincterotomy causes less post-procedure pancreatitis than blended current. Gastrointest Endosc.

[46] Siegel JH, Veerappan A, Tucker R (1994). Bipolar versus monopolar sphincterotomy: a prospective trial. Am J Gastroenterol.

[47] Verma D, Kapadia A, Adler DG (2007). Pure versus mixed electrosurgical current for endoscopic biliary sphincterotomy: a meta-analysis of adverse outcomes. Gastrointest Endosc.

[48] Pasricha P (1998). Current news about sphincterotomy-induced pancreatitis: does electrocautery setting make a difference?. Gastroenterology.

[49] Perini RF, Sadurski R, Cotton PB, Patel RS, Hawes RH, Cunningham JT (2005). Post-sphincterotomy bleeding after the introduction of microprocessor-controlled electrosurgery: does the new technology make the difference?. Gastrointest Endosc.

[50] Mazaki T, Mado K, Masuda H, Shiono M (2014). Prophylactic pancreatic stent placement and post-ERCP pancreatitis: an updated meta-analysis. J Gastroenterol.

[51] Choudhary A, Bechtold ML, Arif M (2011). Pancreatic stents for prophylaxis against post-ERCP pancreatitis: a meta-analysis and systematic review. Gastrointest Endosc.

[52] Mazaki T, Masuda H, Takayama T (2010). Prophylactic pancreatic stent placement and post-ERCP pancreatitis: a systematic review and meta-analysis. Endoscopy.

[53] Afghani E, Akshintala VS, Khashab MA (2014). 5-Fr vs. 3-Fr pancreatic stents for the prevention of post-ERCP pancreatitis in high-risk patients: a systematic review and network meta-analysis. Endoscopy.

[54] Fujisawa T, Kagawa K, Ochiai K (2016). Prophylactic efficacy of 3- or 5-cm pancreatic stents for preventing post-ERCP pancreatitis: a prospective, randomized trial. J Clin Gastroenterol.

[55] Tse F, Yuan Y, Moayyedi P, Leontiadis GI (2012). Guidewire-assisted cannulation of the common bile duct for the prevention of post-endoscopic retrograde cholangiopancreatography (ERCP) pancreatitis. Cochrane Database Syst Rev.

[56] Cennamo V, Fuccio L, Zagari RM (2009). Can a wire-guided cannulation technique increase bile duct cannulation rate and prevent post-ERCP pancreatitis?: a meta-analysis of randomized controlled trials. Am J Gastroenterol.

[57] Kawakami H, Maguchi H, Mukai T (2012). A multicenter, prospective, randomized study of selective bile duct cannulation performed by multiple endoscopists: the BIDMEN study. Gastrointest Endosc.

[58] Kobayashi G, Fujita N, Imaizumi K (2013). Wire-guided biliary cannulation technique does not reduce the risk of post-ERCP pancreatitis: multicenter randomized controlled trial. Dig Endosc.

[59] Mariani A, Giussani A, Di Leo M, Testoni S, Testoni PA (2012). Guidewire biliary cannulation does not reduce post-ERCP pancreatitis compared with the contrast injection technique in low-risk and high-risk patients. Gastrointest Endosc.

[60] Nakai Y, Isayama H, Sasahira N (2015). Risk factors for post-ERCP pancreatitis in wire-guided cannulation for therapeutic biliary ERCP. Gastrointest Endosc.

[61] Chandrasekhara V, Khashab MA, Muthusamy VR (2017). Adverse events associated with ERCP. Gastrointest Endosc.

[62] Radadiya D, Devani K, Arora S, Charilaou P, Brahmbhatt B, Young M, Reddy C (2019). Peri-procedural aggressive hydration for post endoscopic retrograde cholangiopancreatography (ERCP) pancreatitis prophylaxsis: meta-analysis of randomized controlled trials. Pancreatology.

[63] Mäkelä A, Kuusi T, Schröder T (1997). Inhibition of serum phospholipase-A2 in acute pancreatitis by pharmacological agents in vitro. Scand J Clin Lab Invest.

[64] Patai Á, Solymosi N, Mohácsi L, Patai ÁV (2017). Indomethacin and diclofenac in the prevention of post-ERCP pancreatitis: a systematic review and meta-analysis of prospective controlled trials. Gastrointest Endosc.

[65] Andriulli A, Leandro G, Federici T, Ippolito A, Forlano R, Iacobellis A, Annese V (2007). Prophylactic administration of somatostatin or gabexate does not prevent pancreatitis after ERCP: an updated meta-analysis. Gastrointest Endosc.

[66] Wang G, Xiao G, Xu L (2018). Effect of somatostatin on prevention of post-endoscopic retrograde cholangiopancreatography pancreatitis and hyperamylasemia: a systematic review and meta-analysis. Pancreatology.

[67] Katsinelos P, Fasoulas K, Paroutoglou G (2012). Combination of diclofenac plus somatostatin in the prevention of post-ERCP pancreatitis: a randomized, double-blind, placebo-controlled trial. Endoscopy.

[68] Chen B, Fan T, Wang CH (2010). A meta-analysis for the effect of prophylactic GTN on the incidence of post-ERCP pancreatitis and on the successful rate of cannulation of bile ducts. BMC Gastroenterol.

[69] Kubiliun NM, Adams MA, Akshintala VS (2015). Evaluation of pharmacologic prevention of pancreatitis after endoscopic retrograde cholangiopancreatography: a systematic review. Clin Gastroenterol Hepatol.

[70] Sotoudehmanesh R, Eloubeidi MA, Asgari AA, Farsinejad M, Khatibian M (2014). A randomized trial of rectal indomethacin and sublingual nitrates to prevent post-ERCP pancreatitis. Am J Gastroenterol.

[71] Tomoda T, Kato H, Ueki T (2019). Combination of diclofenac and sublingual nitrates is superior to diclofenac alone in preventing pancreatitis after endoscopic retrograde cholangiopancreatography. Gastroenterology.

[72] Yuhara H, Ogawa M, Kawaguchi Y, Igarashi M, Shimosegawa T, Mine T (2014). Pharmacologic prophylaxis of post-endoscopic retrograde cholangiopancreatography pancreatitis: protease inhibitors and NSAIDs in a meta-analysis. J Gastroenterol.

[73] Li S, Cao G, Chen X, Wu T (2012). Low-dose heparin in the prevention of post endoscopic retrograde cholangiopancreatography pancreatitis: a systematic review and meta-analysis. Eur J Gastroenterol Hepatol.

[74] Fluhr G, Mayerle J, Weber E (2013). Pre-study protocol MagPEP: a multicentre randomized controlled trial of magnesium sulphate in the prevention of post-ERCP pancreatitis. BMC Gastroenterol.

[75] Qin X, Lei WS, Xing ZX, Shi F (2015). Prophylactic effect of somatostatin in preventing Post-ERCP pancreatitis: an updated meta-analysis. Saudi J Gastroenterol.

[76] Odes HS, Novis BN, Barbezat GO, Bank S (1977). Effect of calcitonin on the serum amylase levels after endoscopic retrograde cholangiopancreatography. Digestion.

[77] Kaffes AJ, Bourke MJ, Ding S, Alrubaie A, Kwan V, Williams SJ (2006). A prospective, randomized, placebo-controlled trial of transdermal glyceryl trinitrate in ERCP: effects on technical success and post-ERCP pancreatitis. Gastrointest Endosc.

[78] Sudhindran S, Bromwich E, Edwards PR (2001). Prospective randomized double-blind placebo-controlled trial of glyceryl trinitrate in endoscopic retrograde cholangiopancreatography-induced pancreatitis. Br J Surg.

[79] Moretó M, Zaballa M, Casado I (2003). Transdermal glyceryl trinitrate for prevention of post-ERCP pancreatitis: a randomized double-blind trial. Gastrointest Endosc.

[80] Beauchant M, Ingrand P, Favriel JM (2008). Intravenous nitroglycerin for prevention of pancreatitis after therapeutic endoscopic retrograde cholangiography: a randomized, double-blind, placebo-controlled multicenter trial. Endoscopy.

[81] Nøjgaard C, Hornum M, Elkjaer M (2009). Does glyceryl nitrate prevent post-ERCP pancreatitis? A prospective, randomized, double-blind, placebo-controlled multicenter trial. Gastrointest Endosc.

[82] Jowell PS, Branch MS, Fein SH (2011). Intravenous synthetic secretin reduces the incidence of pancreatitis induced by endoscopic retrograde cholangiopancreatography. Pancreas.

[83] Cooper ST, Slivka A (2007). Incidence, risk factors, and prevention of post-ERCP pancreatitis. Gastroenterol Clin North Am.

[84] Sand J, Nordback I (1993). Prospective randomized trial of the effect of nifedipine on pancreatic irritation after endoscopic retrograde cholangiopancreatography. Digestion.

[85] Prat F, Amaris J, Ducot B (2002). Nifedipine for prevention of post-ERCP pancreatitis: a prospective, double-blind randomized study. Gastrointest Endosc.

[86] Gorelick A, Barnett J, Chey W, Anderson M, Elta G (2004). Botulinum toxin injection after biliary sphincterotomy. Endoscopy.

[87] Cotton PB, Hawes RH (2004). Botulinum toxin injection after biliary sphincterotomy. Endoscopy.

[88] Wehrmann T (2004). Sphincter of Oddi dysfunction: cut and inject, but don't measure the pressure?. Endoscopy.

[89] Räty S (2001). Post-ERCP pancreatitis: reduction by routine antibiotics. J Gastrointest Surg.

[90] Lavy A, Karban A, Suissa A, Yassin K, Hermesh I, Ben-Amotz A (2004). Natural beta-carotene for the prevention of post-ERCP pancreatitis. Pancreas.

[91] Gu WJ, Wei CY, Yin RX (2013). Antioxidant supplementation for the prevention of post-endoscopic retrograde cholangiopancreatography pancreatitis: a meta-analysis of randomized controlled trials. Nutr J.

[92] Fan WT, Wang QW, Li QL (2004). [Preventive effect of 5-fluorouracil on post-ERCP pancreatitis]. Zhong Nan Da Xue Xue Bao Yi Xue Ban.

